# A semi-active H∞ control strategy with application to the vibration suppression of nonlinear high-rise building under earthquake excitations

**DOI:** 10.1186/s40064-016-2635-1

**Published:** 2016-07-11

**Authors:** Guiyun Yan, Fuquan Chen, Yingxiong Wu

**Affiliations:** Fujian Provincial Key Laboratory of Advanced Technology and Informatization in Civil Engineering, Department of Civil Engineering, Fujian University of Technology, Fuzhou, 350118 China; College of Civil Engineering, Fuzhou University, Fuzhou, 350116 China

**Keywords:** Semi-active strategy, H∞ control algorithm, High-rise building, Nonlinear seismic response, Kalman–Bucy estimator

## Abstract

Different from previous researches which mostly focused on linear response control of seismically excited high-rise buildings, this study aims to control nonlinear seismic response of high-rise buildings. To this end, a semi-active control strategy, in which H∞ control algorithm is used and magneto-rheological dampers are employed for an actuator, is presented to suppress the nonlinear vibration. In this strategy, a modified Kalman–Bucy observer which is suitable for the proposed semi-active strategy is developed to obtain the state vector from the measured semi-active control force and acceleration feedback, taking into account of the effects of nonlinearity, disturbance and uncertainty of controlled system parameters by the observed nonlinear accelerations. Then, the proposed semi-active H∞ control strategy is applied to the ASCE 20-story benchmark building when subjected to earthquake excitation and compared with the other control approaches by some control criteria. It is indicated that the proposed semi-active H∞ control strategy provides much better control performances by comparison with the semi-active MPC and Clipped-LQG control approaches, and can reduce nonlinear seismic response and minimize the damage in the buildings. Besides, it enhances the reliability of the control performance when compared with the active control strategy. Thus, the proposed semi-active H∞ control strategy is suitable for suppressing the nonlinear vibration of high-rise buildings.

## Background

In the past 20 years, much progress has been made in the field of vibration control of civil structures for the mitigation of earthquake hazard. The previous researches mainly focused on controlling linear response of seismically excited buildings (Dyke et al. 1998; Cai et al. 2000; Mei et al. 2001; Shayeghi et al. 2009; Mohajer et al. 2013). However, structural-member yield may occur during strong ground motions, causing significantly different nonlinear response behavior. Thus, the control strategies designed for suppressing linear response of structures are not appropriate for controlling nonlinear response of structures. In recent years, the control of nonlinear seismic response of structures has been the main concern of structural control research. Ohtori et al. (2004) presented a third structural control benchmark problem which focused on structural control of seismically excited nonlinear structures. This new structural control benchmark problem was different from previous two benchmark problems (Spencer et al. 1998a, b, 1999). The first benchmark problem was about two laboratory scale structures concerning an active control system, and the second further examining the seismic control problem for actual buildings. Given the fact that the two structural control benchmark problems are limited to the linear performance of structures, the third structural control problem provided a common platform, which was to evaluate control devices and the relevant algorithms that command these devices to produce controlling forces, allowing for direct comparison. Li and Ou (2006) examined an adaptive fuzzy sliding-mode control scheme in which magneto-rheological dampers are employed as an actuator to suppress the vibration of the 3-story and 20-story building models. Attard (2007) used viscous dampers, which was commanded by a gradient-based optimization algorithm, to simultaneously control interstory displacements. This method was applied to the 20-story building and was shown to have good performance on controlling the interstory displacement, post-yield curvature, and plastic hinges. Yan et al. (2007) investigated effects of the semi-active model predictive control (MPC) for the 20-story nonlinear building, and the results showed that the proposed semi-active strategy reduced the nonlinear seismic response of high-rise building caused by strong earthquakes. Lei et al. (2012, 2013) applied a decentralized structural control algorithm for active control of the 20-story nonlinear benchmark building, and the results showed that the developed decentralized control provided satisfactory control performances when compared with the conventional centralized control. Cha et al. (2013) conducted a research on optimal placement of active control devices and sensors in the 20-story nonlinear structures using multi-objective genetic algorithms under earthquake loading. Osman and Stefan (2012) employed a new recentering variable friction device (RVFD) to control the seismic response of a 20-story nonlinear benchmark building. To control the vibration of the 20-story nonlinear structure when subjected to earthquake excitation, Li et al. (2011) used fuzzy logic control algorithm to command the hybrid active mass damper (AMD). Yoshida and Dyke (2004) developed a semi-active strategy based on a Clipped-LQG control algorithm which employs absolute acceleration feedback, and this strategy was applied to reduce the structural responses of the 20-story benchmark building. In above studies, the researchers usually define, evaluate and report the performance of their own proposed strategies. However, they do not make a direct comparison to other results.

H∞ control theory is known to offer excellent control performance in dealing with ‘worst-case’ external disturbances, and it can also consider modeling uncertainties. This theory has been successfully applied to civil engineering structures. Chang and Lin (2009) designed an active tendon system in which an optimal H∞ control algorithm was employed to reduce its interstory drift when subjected to earthquake excitation. Yang et al. (2009) designed a decentralized H∞ controller for large-scale civil structures. Jabbari et al. (1995) designed a H∞ controller for seismic-excited buildings with acceleration feedback to reduce both the absolute acceleration and interstory drift. Xiang and Nishitani (2015) explored optimum design of tuned mass damper floor system integrated into bending-shear type building based on H∞, H2, and stability maximization criteria. Rubió-Massegú et al. (2012) presented a new method in designing static output-feedback *H∞* controllers suitable for vibration control of buildings under seismic excitation. Ou et al. (2015) presented the robust integrated actuator control (RIAC) strategy based on *H∞* optimization. In above studies, the researchers mostly concentrated on the active control of linear seismic response using H∞ algorithm.

In this paper, we focus on the control of nonlinear seismic response of high-rise buildings using a semi-active H∞ control strategy when subjected to earthquake excitation. To this end, we develops a novel H∞ controller suitable for semi-active strategies for suppressing nonlinear seismic response of a high-rise building,in which magneto-rheological dampers are employed for an actuator. To estimate the state vector of the H∞ controller from the semi-active control force and acceleration feedback, a modified Kalman–Bucy observer is proposed, which take into account of the effects of nonlinearity, disturbance and uncertainty of controlled system parameters by the observed nonlinear accelerations. Next, a numerical study is conducted to explore the effectiveness of the semi-active H∞ control strategy in suppressing the nonlinear seismic response of a 20-story benchmark structure. Control effects by the proposed strategy are compared with those by the semi-active MPC and Clipped-LQG control approaches. It is found that the proposed control strategy can effectively reduce the nonlinear seismic response of the 20-story benchmark structure.

## Description of nonlinear benchmark building

**Fig. 1:**
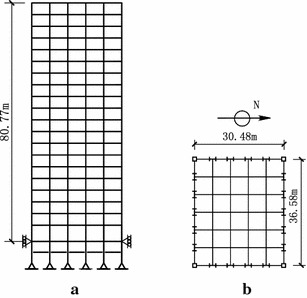
Schematic of 20-story benchmark building. **a** Elevation; **b** plan

As shown in Fig. 1, the building employed herein for control is a 20-story nonlinear benchmark building. It is 36.58 m by 30.48 m in plan and 80.77 m in elevation (Ohtori et al. 2004). The bays are 6.10 m on centre, in both directions, with six bays in the east–west (E–W) direction and five bays in the north–south (N–S) direction. The lateral load-resisting system is composed of steel perimeter moment-resisting frames with composite floors. The columns are 345 MPa steel and the floor system is composed of 248 MPa steel wide-flange beams acting compositely with the floor slab. Assuming that the first two damping ratios are 2 %, the damping matrix is determined on the basis of the assumption of Rayleigh damping. The first five natural frequencies of the twenty-story benchmark building are: 0.261, 0.753, 1.30, 1.83 and 2.40 Hz.

When severe earthquakes occur, structural members may yield and trigger nonlinear responses. To capture the nonlinear behavior, a bilinear hysteresis model is employed to model the plastic hinges which are assumed to occur at the moment resisting column–beam and column–column connections in the 20-story building. The bilinear bending properties are predefined for each structural member. A material nonlinear behavior in the structure is taken into account by these plastic hinges.

Apart from the seventeen evaluation criteria as shown Table 1, another two criteria are considered to depict the performance of the controlled system (Yoshida and Dyke 2004). These two criteria are dimensionless parameters which characterize the maximum and the total permanent interstory drift caused by the formation of plastic hinges after severe earthquakes, and defined as1where *θ*_*pi*_ = |*d*_*pi*_|/*h*_*i*_, *d*_*pi*_ and *h*_*i*_ denote the permanent interstory drift and the height of the *i*-th floor of the controlled structures. *θ*_*p*_^max^ and *θ*_*p*_^*sum*^ are the maximum and the total permanent interstory drift ratio of the uncontrolled structure.

**Table 1:**
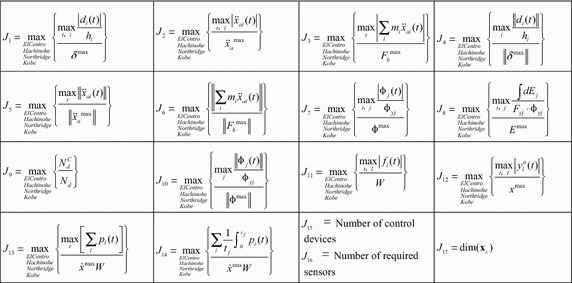
Summary of evaluation criteria for the nonlinear benchmark problem

As shown in Table 1, *d*_*i*_(*t*) and $\ddot{x}_{ai}(t)$ represent the seismic interstory drift and the absolute acceleration response of the *i*-th floor of controlled structures; *ϕ*_*j*_ denote the dissipated energy by plastic hinges in the member during the each earthquake. Other parameters are further depicted in the benchmark statement paper (Ohtori et al. 2004).

## Mechanical model for control devices

To control nonlinear seismic response of the 20-story benchmark structure, magneto-rheological dampers are employed as control devices. A simple Bingham plasticity model can effectively describe the essential field dependent fluid characteristic. In this model, the total shear stress is expressed as (Yang et al. 2002)

2a $$\tau = \tau_{y} (H)\text{sgn} (\dot{\gamma }) + \eta \dot{\gamma }\quad \left| \tau \right| > \left| {\tau_{y} } \right|$$

2b $$\dot{\gamma } = 0\quad \left| \tau \right| \le \left| {\tau_{y} } \right|$$

where *τ*_*y*_ and $\dot{\gamma }$ are yield stress resulting from the applied magnetic field and shear strain rate, respectively. *H* denote amplitude of the applied field; and *η* represent field-independent post-yield plastic viscosity.

On the basis of the proposed and validated parallel-plate model (Zhou et al. 2001), the damper resisting force can be decomposed into an uncontrollable force *F*_*η*_ and a controllable force *F*_*τ*_ owing to controllable yield stress *τ*_*y*_:

3a $$F(t) = F_{\eta } (t) + F_{\tau } (t)$$

3b $$F_{\eta } (t) = \frac{{12\eta LA_{p} }}{{\pi Dh^{3} }}A_{p} \dot{v}(t);\quad F_{\tau } (t) = \frac{{3L\tau_{y} }}{h}A_{p} \text{sgn} \left[ {\dot{v}\left( t \right)} \right]$$

3c $$A_{P} = \frac{\pi }{4}\left( {D_{0}^{2} - d^{2} } \right)$$

The meaning of parameters is explained in detail (Zhou et al. 2001). To conveniently compare the analysis results of different control strategies with application to seismically excited nonlinear buildings, the magneto-rheological dampers herein are also designed to have maximum capacity of 1000 kN with maximum command voltage V_max_ = 10 V, in consistence with those dampers described in the literatures (Yoshida and Dyke 2004; Yan 2006).

## The proposed semi-active H∞ control strategy

Consider a seismically excited nonlinear building modeled by an *n*-degrees-of freedom system and controlled with *r* control devices. The motion equations can be written as

4 $${\mathbf{M}}{\ddot{\mathbf{x}}}(t) + {\mathbf{C}}{\dot{\mathbf{x}}}(t) + {\mathbf{K}}[{\mathbf{x}}(t)]{\mathbf{x}}(t) = {\mathbf{\varLambda f}}(t) - {\mathbf{\varGamma w}}(t)$$

where **x**(*t*), ${\dot{\mathbf{x}}}(t)$, and ${\ddot{\mathbf{x}}}(t)$ is the *n*-dimensional displacement, velocity, and acceleration vector, respectively; **M**, **C**, and **K**[**x**(*t*)] are the *n* × *n* mass, damping and nonlinear stiffness matrices, respectively; and **w**(*t*) is the one-dimensional disturbance vector with influence matrix Γ, representing the loading due to earthquake ground motion. **f**(*t*) is the *r*-dimensional vector of control force generated by the control devices with location matrix Λ.

Represented in state-space form, Eq. (4) can be rewritten as

5 $${\dot{\mathbf{X}}}(t) = {\mathbf{AX}}(t) + {\mathbf{Bf}}(t) + {\mathbf{Ew}}(t)$$

where

$$\begin{aligned}{\mathbf{X}}(t) &= \left[{\begin{array}{*{20}l} {{\mathbf{x}}(t)} \\ {{\dot{\mathbf{x}}}(t)} \\ \end{array}} \right];\quad {\mathbf{A}} = \left[{\begin{array}{*{20}c} O &\quad {\mathbf{I}} \\ {- {\mathbf{M}}^{- 1} {\mathbf{Kx}}(t)} &\quad {- {\mathbf{M}}^{- 1} {\mathbf{C}}} \\ \end{array}} \right]\\ {\mathbf{B}} &= \left[ {\begin{array}{*{20}c} O \\ {{\mathbf{M}}^{ - 1} {\varvec{\Lambda}}} \\ \end{array} } \right];\quad{\mathbf{E}} = \left[ {\begin{array}{*{20}c} O \\ { - {\mathbf{M}}^{ - 1} {\varvec{\Gamma}}} \\ \end{array} } \right] \end{aligned}$$

**X**(*t*) is a 2*n* × 1 state vector, **A** is a 2*n* × 2*n* system matrix, **B** is a 2*n* × *r* controller location matrix, and **E** is a 2*n* × 1 external excitation location matrix, respectively. Define a *p* × 1 control output vector **Z**(*t*) as

6 $${\mathbf{Z}}({\mathbf{t}}) = {\mathbf{C}}_{1} {\mathbf{X}}({\mathbf{t}}) + {\mathbf{Df}}({\mathbf{t}})$$

where **C**_1_ and **D** are *p* × 2*n* and *p* × *r* matrices.

### Full state feedback H∞ control

The reduced order building model with twenty states have been developed for purposes of control design by Ohtori et al. (2004). This model is still adopted in this study. Then the full state feedback control forces are determined by

7 $${\mathbf{f}}({\mathbf{t}}) = {\mathbf{GX}}({\mathbf{t}})$$

where **G** is called the control gain matrix. Replacing **f**(*t*) with **GX**(*t*) in Eqs. (5) and (6), the state-space equations can be written as

8a $${\dot{\mathbf{X}}}(t) = {\mathbf{A}}_{\text{CL}} {\mathbf{X}}(t) + {\mathbf{Ew}}(t)$$

8b $${\mathbf{Z}}(t) = {\mathbf{C}}_{\text{CL}} {\mathbf{X}}(t)$$

where

$${\mathbf{A}}_{\text{CL}} = {\mathbf{A}} + {\mathbf{BG}}$$

$${\mathbf{C}}_{\text{CL}} = {\mathbf{C}}_{1} + {\mathbf{DG}}$$

In frequency domain, the dynamic systems can be represented by the transfer function from disturbance **w**(*t*) to output **Z**(*t*) as

9 $${\mathbf{H}}_{{{\mathbf{Zw}}}} (s) = {\mathbf{C}}_{\text{CL}} (s{\mathbf{I}} - {\mathbf{A}}_{CL} )^{ - 1} {\mathbf{\rm E}}$$

where *s* is the complex Laplaceian variable.

By minimizing the H∞-norm of the closed-loop system, H∞ control in the frequency domain is given by

10 $$\left\| {{\mathbf{T}}_{{{\mathbf{Zw}}}} (s)} \right\|_{\infty } \mathop = \limits^{\Delta } \mathop {\sup }\limits_{\omega } \overline{\sigma } \left[ {{\mathbf{T}}_{{{\mathbf{Zw}}}} (j\omega )} \right] \le \delta$$

where *δ* denotes a positive attenuation constant which is a measure of control performance. To achieve more strict performance of the control system, a smaller value of *δ* is required. $\bar{\sigma }\left[ \cdot \right]$ is the largest singular value of a matrix, and ‘sup’ represents the supremum of a set of real numbers, *ω* represents angular frequency, *j* denotes the imaginary unit. The definition indicates that the H∞-norm of the system in the frequency domain equals to the peak of the largest singular value of the transfer function **T**_*Zw*_(*s*) along the imaginary axis. Also, the H∞-norm has an equal meaning in the time domain, for the supremum of the 2-norm amplification from the disturbance to the output:

11 $$\left\| {{\mathbf{T}}_{{{\mathbf{Zw}}}} (s)} \right\|_{\infty } \mathop = \limits^{\Delta } \mathop {\sup }\limits_{{{\mathbf{W}},\left\| {{\mathbf{W}}\left( t \right)} \right\|_{2} \ne 0}} \left( {\left\| {{\mathbf{Z}}(t))} \right\|_{2} /\left\| {{\mathbf{w}}(t)} \right\|_{2} } \right)$$

where the 2-norm of a signal **v**(*t*) is expressed as $\left\| {{\mathbf{v}}(t)} \right\|_{2} = \sqrt {\int_{t = - \infty }^{t = + \infty } {{\mathbf{v}}^{T} (t){\mathbf{v}}(t)dt} }$. The H∞-norm herein can be regarded as the upper limit of the application factor from the disturbance (i.e. seismic ground motion) energy to the output (i.e. structural response) energy. When this upper limit is reached, the disturbance is called a ‘worst-case’ disturbance. At the same time, the system output with structural response can be significantly reduced by minimizing the H∞-norm.

The norm of controlled output (6) that includes cross-product terms in **X**(*t*) and **f**(*t*) is considered in this paper; i.e. the orthogonality condition **D**^*T*^**C**_1_ = 0 does not hold. By appropriate scaling of **f**(*t*), the assumption that **D** is full rank and **D**^T^**D** = **I** can always be satisfied. The following result presented by Jabbari et al. (1995) is applied to this problem.

Lemma: Consider the system in (5) with the control output (6), where **D**^T^**C**_1_ ≠ 0 and **D**^T^**D** = **I**. For a given *δ* > 0, if there exists a positive definite solution **P** to

12 $$\begin{aligned} &{\mathbf{P}}\left( {{\mathbf{A}} - {\mathbf{BD}}^{T} {\mathbf{C}}_{1} } \right) + \left( {{\mathbf{A}} - {\mathbf{BD}}^{T} {\mathbf{C}}_{1} } \right)^{T} {\mathbf{P}} + {\mathbf{Q}} \\&\quad - {\mathbf{P}}\left( {{\mathbf{BB}}^{T} - \frac{1}{{\delta^{2} }}{\mathbf{EE}}^{T} } \right){\mathbf{P}} \, + {\mathbf{C}}_{1}^{T} \left( {{\mathbf{I}} - {\mathbf{DD}}^{T} } \right){\mathbf{C}}_{1} = 0 \end{aligned}$$

for some positive definite matrix **Q** > 0, then the control law

13 $${\mathbf{f}}(t) = - \left( {{\mathbf{B}}^{T} {\mathbf{P}} + {\mathbf{D}}^{T} {\mathbf{C}}_{1} } \right){\mathbf{X}}(t)$$

is a stabilizing control law for (5), and $\left\| {{\mathbf{T}}_{{_{{{\mathbf{Zw}}}} }} (s)} \right\|_{\infty } \le \delta$.

### Semi-active H∞ control strategy

Different from the standard H∞ method, the control design process in this paper can follow two steps. Firstly, the control law can be designed on the basis of full state feedback. Next, a modified Kalman–Bucy observer suitable for semi-active strategy can be designed based on the control gains obtained in the first step. Therefore, we focus on the trade-offs about going from full state feedback to observer based on controllers.

Because the evaluation model in the third generation benchmark control problem for the 20-story nonlinear building is quite large, the relatively accuracy reduced-order building model which is used for designing the controller is obtained by the Guyan-State reduction suggested by Yan (2006). In design of the controller, this model is also adopted in this paper. This 20-state reduced order model of the 20-story building is represented as

14 $${\dot{\mathbf{x}}}_{c} = {\mathbf{A}}_{c} {\mathbf{x}}_{c} + {\mathbf{B}}_{c} {\mathbf{f}} + {\mathbf{E}}_{c} {\ddot{\mathbf{x}}_\mathbf{g}}$$

where **x**_*c*_ is the design state vector, and **A**_*c*_, **B**_*c*_, **E**_*c*_ are the reduced order coefficient matrices.

A controlled output vector is written as

15 $${\mathbf{z}} = {\mathbf{C}}_{z} {\mathbf{x}}_{c} + {\mathbf{D}}_{z} {\mathbf{f}}$$

where **C**_*z*_ and **D**_*z*_ are the reduced order coefficient matrices.

The control **f** is included in the **z**-vector to allow penalizing large control input forces. For the multi-input systems, we can use appropriate scaling factors *α*_*i*_ to weight on the control forces. In this case, by increasing *α*_*i*_, we can decrease the weigh on the actual control and put more emphasis on the states through **C**_*z*_**x**_*c*_. Thus, the coefficient matrices **B**_*c*_ and **D**_*z*_ can be modified as follows

16 $${\hat{\mathbf{B}}}_{c} = \alpha_{i} {\mathbf{B}}_{cji};{\hat{\mathbf{D}}}_{z} = \alpha_{i} {\mathbf{D}}_{zji} \quad i = 1,2 \ldots,r;\;\;j = 1,2, \ldots,2n$$

17 $$\hat{f}_{i} = \frac{1}{{\alpha_{i} }}f_{i}$$

18 $${\mathbf{f}} = [f_{1},f_{2}, \ldots,f_{r}]^{\text{T}};\quad {\hat{\mathbf{f}}} = [\hat{f}_{1},\hat{f}_{2}, \ldots, \hat{f}_{r}]^{\text{T}}$$

Therefore, the state space forms for control design are rewritten as

19 $$ {\dot{\mathbf{x}}}_{c} = {\mathbf{A}}_{c} {\mathbf{x}}_{c} + {\hat{\mathbf{B}}}_{c} {\hat{\mathbf{f}}} + {\mathbf{E}}_{c} {\ddot{\mathbf{x}}_\mathbf{g}} $$

20 $${\mathbf{z}} = {\mathbf{C}}_{z} {\mathbf{x}}_{c} + {\hat{\mathbf{D}}}_{z} {\hat{\mathbf{f}}}$$

The Riccati equation for Eqs. (19) and (20) is expressed as

21 $$\begin{aligned} &{\mathbf{P}}_{c} \left( {{\mathbf{A}}_{c} - {\hat{\mathbf{B}}}_{c} {\hat{\mathbf{D}}}_{z}^{T} {\mathbf{C}}_{z} } \right) + \left( {{\mathbf{A}}_{c} - {\hat{\mathbf{B}}}_{c} {\hat{\mathbf{D}}}_{z}^{T} {\mathbf{C}}_{z} } \right)^{T} {\mathbf{P}}_{c} + {\mathbf{Q}}\\&\quad- {\mathbf{P}}_{c} \left( {{\hat{\mathbf{B}}}_{c} {\hat{\mathbf{B}}}_{c}^{T} - \frac{1}{{\delta^{2} }}{\mathbf{E}}_{c} {\mathbf{E}}_{c}^{T} } \right){\mathbf{P}}_{c} + {\mathbf{C}}_{z}^{T} \left( {{\mathbf{I}} - {\hat{\mathbf{D}}}_{z} {\hat{\mathbf{D}}}_{z}^{T} } \right){\mathbf{C}}_{z} = 0 \end{aligned}$$

For a given *δ* > 0 and **Q** > 0, if there exists a positive definite solution **P**_*c*_ to Eq. (21), then the *r*-dimensional control vector ${\hat{\mathbf{f}}}$ are expressed as

22 $${\hat{\mathbf{f}}} = - {\mathbf{Gx}}_{c} = - \left( {{\hat{\mathbf{B}}}_{c}^{T} {\mathbf{P}}_{c} + {\hat{\mathbf{D}}}_{z}^{T} {\mathbf{C}}_{z} } \right){\mathbf{x}}_{c}$$

The application of the controller in Eq. (22), however, requires the measurement of the all state vector, which may be impractical. In the following section, the H∞ control technique is extended to contain the ability of using sensors that measure limited number of floor accelerations for direct measurements of floor accelerations is most reliable.

We consider a measured output vector of limited number of floor accelerations

23 $$ {\mathbf{y}}_{m} = {\mathbf{C}}_{m}^{{}} {\mathbf{x}}_{c} + {\mathbf{D}}_{m}^{{}} {\mathbf{f}} + {\mathbf{E}}_{m}^{{}} {\ddot{\mathbf{x}}_\mathbf{g}} $$

24 $$ {\mathbf{y}}_{m} = {\mathbf{C}}_{m} {\mathbf{x}}_{c} + {\hat{\mathbf{D}}}_{m} {\hat{\mathbf{f}}} + {\mathbf{E}}_{m} {\ddot{\mathbf{x}}_\mathbf{g}} $$

25 $${\hat{\mathbf{D}}}_{m} = \alpha_{i} {\mathbf{D}}_{mji}$$

where **v** is a disturbance vector of measurement noise, and **C**_*m*_, **D**_*m*_, **E**_*m*_ are the reduced order coefficient matrices.

Based on the measured vectors of floor accelerations and semi-active control forces, a modified Kalman–Bucy observer suitable for semi-active strategy is established to obtain the estimation of the state vector. It can be expressed as

26 $${\dot{\hat{\mathbf{x}}}}_{c} = \left( {{\mathbf{A}}_{c} - {\mathbf{LC}}_{m} } \right){\hat{\mathbf{x}}}_{c} + {\mathbf{Ly}}_{m} + \left( {{\hat{\mathbf{B}}}_{c} - {\mathbf{L}}{\hat{\mathbf{D}}}_{m} } \right){\hat{\mathbf{f}}}_{semi}$$

27 $$\begin{aligned} {\hat{\mathbf{f}}}_{{semi}} & = [\hat{f}_{{semi(1)}} ,\hat{f}_{{semi(2)}} {\text{,}} \ldots {\text{,}}\hat{f}_{{semi(r)}} ]^{{\text{T}}} \\ & = \left[ {\begin{array}{*{20}l} {1/\alpha _{1} } \hfill & {} \hfill & {} \hfill \\ {} \hfill & \ddots \hfill & {} \hfill \\ {} \hfill & {} \hfill & {1/\alpha _{r} } \hfill \\ \end{array} } \right]\left[ {\begin{array}{*{20}c} {f_{{semi(1)}} } \\ \vdots \\ {f_{{semi(r)}} } \\ \end{array} } \right] \\ \end{aligned}$$

where ${\hat{\mathbf{x}}}_{c}$ is the estimate for the state vector **x**_*c*_, **L** is the gain matrix of Kalman–Bucy observer, **f**_*semi*_ is the vector of measured control forces generated by magneto-rheological dampers, **y**_*m*_ is the nonlinear response of measured floor accelerations, which considers the effects of nonlinearity and uncertainty of controlled system.

Thus the *r*-dimensional control vector ${\hat{\mathbf{f}}}$ can be rewritten as

28 $${\hat{\mathbf{f}}} = - {\mathbf{G}}{\hat{\mathbf{x}}}_{c} = - \left({\hat{\mathbf{B}}}_{c}^{T} {\mathbf{P}}_{c} + {\hat{\mathbf{D}}}_{z}^{T} {\mathbf{C}}_{z} \right){\hat{\mathbf{x}}}_{c}$$

To implement H∞ control, magneto-rheological dampers are employed as actuators and the controllable damping force of the dampers at time *t* is determined by the control algorithm on the condition that if the force is not dissipative, the magneto-rheological dampers are driven to perform as simple friction dampers. In addition, there is a limitation on the maximum force that the magneto-rheological dampers exert. Thus, the semi-active control strategy should be expressed as

29 $$\begin{aligned} f_{semi(i)} & = \alpha_{i} \cdot \hat{f}_{semi(i)} \\ & = \alpha_{i} \cdot \left\{ {\begin{array}{*{20}l} {\frac{1}{{\alpha_{i} }}F_{\text{max} } ,} \hfill & {{\text{if}}\;\hat{f}_{i} \cdot f_{semi(i)} > 0\;\;{\text{and}}\quad\left| {\hat{f}_{i} } \right| \ge \frac{1}{{\alpha_{i} }}F_{\text{max} } } \hfill \\ {\hat{f}_{i} ,} \hfill & {{\text{if}}\;\hat{f}_{i} \cdot f_{semi(i)} > 0\;\;{\text{and}}\quad\left| {\hat{f}_{i} } \right| < \frac{1}{{\alpha_{i} }}F_{\text{max} } } \hfill \\ {\frac{1}{{\alpha_{i} }}F_{\text{min} } ,} \hfill & {{\text{if}}\;\hat{f}_{i} \cdot f_{semi(i)} \le 0} \hfill \\ \end{array} } \right.\quad i = 1, \ldots ,r \\ \end{aligned}$$

where *f*_*semi*(*i*)_ denotes the actuator force generated by the *i*-th magneto-rheological damper; $\hat{f}_{i}$ is the *i*-th element of ${\hat{\mathbf{f}}}$. *F*_min_ and *F*_max_ are the minimum and maximum damping forces of all magneto-rheological dampers. The control law described in Eq. (29) represents a semi-active H∞ control strategy (semi-active H_infinite_).

## Numerical results

The controllers proposed herein are evaluated by considering the time histories of the controlled structure provided in the benchmark problem. The full model of the structural system, which involves member nonlinearity (Ohtori et al. 2004; Spencer et al. 1999), is used to conduct the simulation.

For design purposes, it is assumed that the measurement noise vector, v, contains identically distributed, statically independent Gaussian white noise process, with $S_{{\ddot{x}_{g} \ddot{x}_{g} }} /S_{{\nu _{i} \nu _{i} }} = \gamma _{g} = 25$. The results of parameters analysis show that an effective controller can be designed by choosing a state vector x_*c*_ to include the displacements and velocities of some floors relative to the ground, i.e., ${\mathbf{x}}_{c} = \left[ {x_{2} ,x_{4} , \ldots ,x_{20} ,\dot{x}_{2} ,\dot{x}_{4} , \ldots ,\dot{x}_{20} } \right]^{\text{T}}$, by selecting a output vector, z to include the accelerations of some floors relative to the ground, i.e., ${\mathbf{z}} ={[\ddot{a}_4,\ddot{a}_8,\ddot{a}_{12},\ddot{a}_{16},\ddot{a}_{20}]^T}$, and by choosing a vector of measured responses to include the absolute accelerations of some floors, *i*.*e*., ${\mathbf{y}}_{m} = [\ddot{x}_4, \ddot{x}_8,\ddot{x}_{12},\ddot{x}_{20}]^T$. The devices installed on each floor from the first to the eighth, the ninth to the seventeen, and the eighteen to the twentieth floor, are four, three and two magneto-rheological dampers respectively, and Q = *q*·*diag*(*I*_1×20_). The other parameters are selected as *α*_1_ ∼ *α*_5_ = 5 × 10^5^, *α*_6_ ∼ *α*_20_ = 8 × 10^5^, *δ* = 5, *q* = 0.1.

**Fig. 2:**
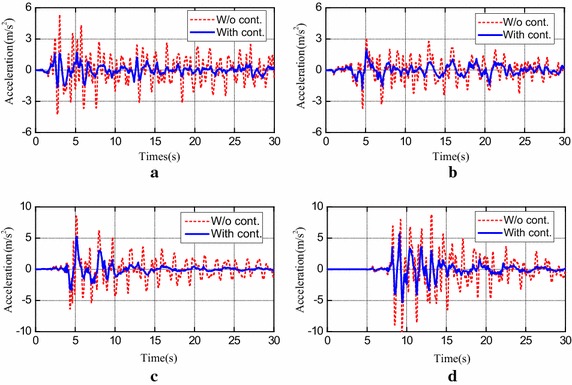
Absolute acceleration responses at 20th floor of building with semi-active H∞ control and without control subjected to different earthquakes. **a** El Centro; **b** Hachinohe; **c** Northridge; **d** Kobe

**Fig. 3:**
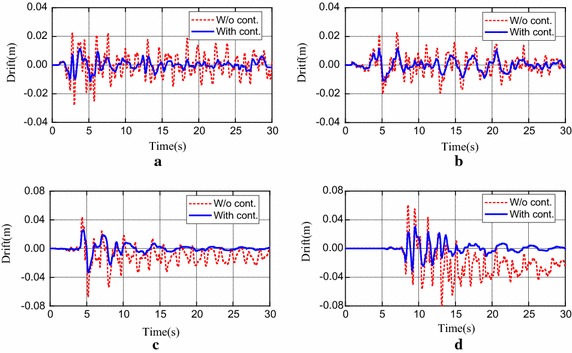
Interstory drift responses between 19th and 20th floors of building with semi-active H∞ control and without control subjected to different earthquakes. **a** El Centro; **b** Hachinohe; **c** Northridge; **d** Kobe

**Fig. 4:**
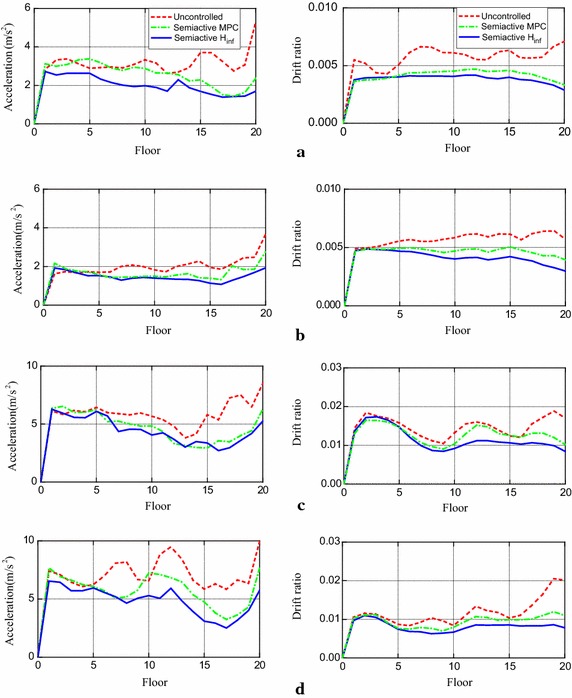
Distribution of peak acceleration and peak interstory drift ratio along height of building with semi-active H∞ control and without control subjected to different earthquakes. **a** El Centro; **b** Hachinohe; **c** Northridge; **d** Kobe

Typical responses of the controlled systems when subjected to the original intensity earthquakes are selected. Absolute acceleration responses at the 20th floor are provided in Fig. 2 and interstory drift responses between the 19th and 20th floors are depicted in Fig. 3. These responses are chosen for the maximum drifts often occur at the 20th floor. Additionally, the distribution of the peak interstory drift ratio and peak acceleration along the building height subjected to different earthquakes are depicted in Fig. 4.

From these results in Figs. 2 and 3, it is observed that both the peak accelerations and peak interstory drifts are greatly decreased when the semi-active H∞ control strategy is adopted to control the structure. Also, in the case of strong earthquake, for instance, the original intensity Northridge and Kobe, great permanent drifts are generated in the building without control. This is because of the development of plastic connections. On the contrary, these permanent drifts are suppressed when the semi-active H∞ control strategy is used.

As shown in Fig. 4, when the semi-active H∞ and semi-active MPC scheme are applied to control the structure, both peak acceleration and peak interstory drift are greatly decreased except in a few cases, where the peak acceleration at a particular floor may have a minor increase. It is worth noting that the proposed semi-active H∞ control strategy gains better control effects when compared with the semi-active MPC scheme.

**Fig. 5:**
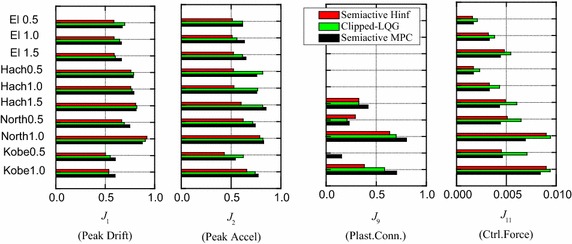
*Bar chart* comparing the evaluation criteria for various controllers

Table 2 lists the values of the evaluation criteria for semi-active H∞ control, semi-active MPC (Yan 2006) and Clipped-LQG (Yoshida and Dyke 2004) systems subjected to different earthquakes with different intensity levels. To compare the efficiency of the three controlled strategies, Fig. 5 provides the contrast of the values for the maximum interstory drift ratio (*J*_1_), maximum absolute acceleration (*J*_2_), number of plastic hinges (*J*_9_) and maximum control force (*J*_11_). These values are derived from the various control systems for different earthquakes with different intensity levels.

**Table 2:** Evaluation criteria for evaluated control systems

Earthquake intensity	Semi-active H∞					Semi-active MPC					Clipped-LQG control				
	El Centro	Hachi.	Northrdg.	Kobe	Max value	El Centro	Hachi.	Northrdg.	Kobe	Max value	El Centro	Hachi.	Northrdg.	Kobe	Max value
	0.5/1.0/1.5	0.5/1.0/1.5	0.5/1.0	0.5/1.0		0.5/1.0/1.5	0.5/1.0/1.5	0.5/1.0	0.5/1.0		0.5/1.0/1.5	0.5/1.0/1.5	0.5/1.0	0.5/1.0	
*J*_1_ Peak drift ratio	0.5870 0.5883 0.5896	0.7581 0.7576 0.8058	0.6648 0.9209	0.5016 0.5360	0.9209	0.6704 0.6628 0.6632	0.7832 0.7891 0.8114	0.7487 0.8741	0.5996 0.5990	0.8741	0.6957 0.6450 0.6007	0.7867 0.7707 0.8180	0.691 0.906	0.5495 0.5324	0.906
*J*_2_ Peak acceleration	0.5129 0.5093 0.5235	0.5235 0.5275 0.5996	0.6208 0.7866	0.4315 0.6561	0.7866	0.617 0.6334 0.6493	0.7572 0.7561 0.8518	0.7427 0.8266	0.5411 0.77	0.9271	0.6149 0.5568 0.6156	0.8171 0.7673 0.8175	0.7167 0.8195	0.6228 0.741	0.8195
*J*_3_ Peak base shear	0.7061 0.7063 0.8266	0.9923 0.9884 1.0194	0.9320 1.0302	0.7264 1.0430	1.0430	0.857 0.8197 0.9306	1.0907 1.0466 1.0733	0.9373 1.0136	0.9629 1.0378	1.0907	0.8145 0.8283 0.9612	1.0519 1.0507 1.0623	1.0019 1.0777	0.766 1.0793	1.0793
*J*_4_ Norm drift ratio	0.5247 0.5232 0.5277	0.7512 0.7526 0.7697	0.4862 1.0809	0.3771 0.1427	1.2809	0.569 0.577 0.5866	0.7928 0.7994 0.8198	0.5814 1.0175	0.4762 0.1867	1.0175	0.5568 0.5261 0.5206	0.7761 0.7553 0.7649	0.4557 1.1881	0.3563 0.1716	1.1881
*J*_5_ Norm acceleration	0.3255 0.3251 0.3351	0.4571 0.4576 0.4689	0.3508 0.4513	0.3080 0.4448	0.4689	0.4786 0.4815 0.4328	0.7025 0.6348 0.5525	0.4739 0.5401	0.5342 0.5717	0.7025	0.5656 0.4833 0.462	0.6647 0.59 0.5699	0.4043 0.4909	0.4237 0.548	0.6647
*J*_6_ Norm base shear	0.5865 0.5847 0.5889	0.7456 0.7462 0.7580	0.5146 0.7295	0.4545 0.6498	0.7580	0.6693 0.6516 0.6568	0.7946 0.7749 0.7848	0.6261 0.7984	0.5845 0.7626	0.7984	0.6733 0.6303 0.6179	0.823 0.784 0.7831	0.5091 0.7429	0.4395 0.6352	0.823
*J*_7_ Ductility	0.6839 0.66847 0.6399	0.9310 0.9303 0.8969	0.7174 0.9423	0.4193 0.5606	0.9423	0.6747 0.6689 0.6253	0.9245 0.9231 0.8789	0.6603 0.9012	0.5763 0.6818	0.9245	0.6992 0.7098 0.6533	0.9569 0.9367 0.8999	0.6837 0.9276	0.4292 0.4985	0.9569
*J*_8_ Dissipated energy	– – 0	– – 0.2548	0.0561 0.3065	0 0.0983	0.3065	– – 0	– – 0.2422	0.0254 0.3936	0.024 0.1109	0.3936	– – 0	– – 0.2567	0.0351 0.2546	0 0.0719	0.2567
*J*_9_ Plastic connection	– – 0	– – 0.3256	0.2917 0.6354	0 0.3810	0.6354	– – 0	– – 0.4186	0.2292 0.8021	0.1538 0.7024	0.8021	– – 0	– – 0.3256	0.2083 0.6979	0 0.5833	0.6979
*J*_10_ Norm ductility	0.5847 0.5832 0.5207	0.7350 0.7360 0.7990	0.3976 1.2573	0.3871 0.1447	1.2573	0.6324 0.6426 0.5793	0.7632 0.7716 0.8125	0.4821 1.0805	0.4919 0.2522	1.0805	0.6136 0.5875 0.5137	0.7519 0.7288 0.8343	0.3858 1.2044	0.3394 0.207	1.2044
*J*_11_ Control force	0.0016 0.0032 0.0048	0.0017 0.0033 0.0049	0.0051 0.0090	0.0045 0.0090	0.0090	0.0017 0.0033 0.0044	0.0017 0.0033 0.0043	0.0044 0.0069	0.0046 0.0084	0.0084	0.002092 0.00382 0.005435	0.002315 0.004334 0.006041	0.00647 0.009429	0.007067 0.009408	0.009429
*J*_12_ Device stroke	0.0680 0.0680 0.0687	0.0746 0.0746 0.0792	0.0792 0.1036	0.0947 0.1030	0.1036	0.0667 0.0655 0.0661	0.0739 0.0737 0.0781	0.0724 0.0965	0.1113 0.1096	0.1113	0.07083 0.07125 0.07095	0.07562 0.07395 0.07793	0.07372 0.09833	0.08558 0.08767	0.09833
*J*_13_ Control power	0.000065 0.000033 0.000023	0.000066 0.000033 0.000024	0.000018 0.000015	0.000023 0.000016	0.000066	0.000065 0.000033 0.000023	0.000066 0.000033 0.000024	0.000018 0.000015	0.000023 0.000016	0.000066	0.000009 0.000009 0.00001	0.000011 0.000011 0.000012	0.000009 0.00001	0.000011 0.000013	0.000013
*J*_14_ Norm control power	0.000065 0.000033 0.000023	0.000066 0.000033 0.000024	0.000018 0.000015	0.000023 0.000016	0.000066	0.000065 0.000033 0.000023	0.000066 0.000033 0.000024	0.000018 0.000015	0.000023 0.000016	0.000066	0.000001 0.000001 0.000001	0.000001 0.000001 0.000001	0 0	0 0	0.000001
*J*_15_ Control devices	65				65	65				65	65				65
*J*_16_ Sensors	25				25	5				5	25				25
*J*_17_ Computational resolution	20				20	20				20	20				20
*J*_18_ Maximum permanent drift	– – 0	– – 0.8389	0.1845 1.0926	0 0.0225	1.0926	– – 0.0022	– – 0.8338	0.0308 1.028	0.0779 0.0902	1.028	– – 0	– – 1.3284	0.1438 1.2149	0 0.1398	1.3284
*J*_19_ Total permanent drift	– – 0	– – 0.4006	0.0832 0.7249	0 0.0174	0.7249	– – 0.0037	– – 0.5005	0.0152 0.7468	0.0292 0.1334	0.7468	– – 0	– – 0.7127	0.0598 0.7214	0 0.1057	0.7214

^a^Note that uncontrolled structure does not yield in some cases such as 0.5/1.0 scale El Centro earthquake and 0.5/1.0 scale Hachi. earthquake

From the first graph in Fig. 5, it is seen that when the semi-active H∞ control strategy is applied, the peak drift ratios of the structure are reduced to 50–60 % of those uncontrolled values for each intensity level of the Kobe and El Centro earthquakes. For the Hachinohe and Northridge earthquakes at all intensity levels, except full-scale Northridge, this proposed strategy reduces the responses to 60–80 %, resulting in a modest reduction, and behaves a little better than the other two schemes.

The comparison for the peak acceleration of the structure are listed in the second graph in Fig. 5. It is observed that all those controllers can reduce the peak acceleration of the uncontrolled structure under all earthquakes with different intensity levels, and the semi-active H∞ controller functions better than the other two controllers. In addition, there is no general tendency when comparing the efficiency for the semi-active MPC and Clipped-LQG controllers.

As shown in the third graph in Fig. 5, it is worth noting that the number of plastic hinges is significantly reduced when a controller is applied. For example, the uncontrolled structure yields plastic hinges under the half-scale intensity Kobe earthquake and the 1.5 scale intensity El Centro earthquake. However, the formation of plastic hinges is completely prevented when the semi-active H∞ control strategy is applied. The same is also observed for the Clipped-LQG controller. In addition, in all the cases that plastic hinges are formed in the uncontrolled structure, the number of plastic hinges is greatly reduced when the proposed control strategy is applied, and the control effects of the proposed strategy are a little better than those by the other two approaches, except the half-scale Northridge case. Therefore, damage in the structure is greatly decreased. As is evident in the drift response time histories of the in Fig. 3, when the structure yields plastic hinges, a permanent deformation remains in the structures. The degree of residual permanent deformation could be indirectly controlled by decreasing the drifts of the structure throughout the earthquake.

In Fig. 5, note that the force requirements of both the semi-active H∞ control and semi-active MPC systems are of similarity for a given earthquake and magnitude, except full-scale intensity Kobe earthquake. In addition, the maximum control forces (*J*_11_) for the semi-active H∞ control and semi-active MPC systems are less than those of the Clipped-LQG control system. It is worth noting that these semi-active systems are inherently stable for they don’t input a large amount of energy into the structural system. Hence, according to stability, the semi-active systems are considerably more robust than the active system.

Values for the additional evaluation criteria are listed in Table 2. Note that all the values of *J*_19_ of the three strategies in all cases are less than 1.0, it indicates that the total permanent deformations in the controlled structure are smaller than those in the uncontrolled structure. In addition, except one case, the value of *J*_18_ is less than 1.0 for the semi-active H∞ control strategy, indicating that the maximum permanent drifts remained in the controlled structure are smaller than those in the uncontrolled structure. Under the full-scale intensity Northridge earthquake, the value of *J*_18_ is above 1.0, which means that the maximum permanent interstory drift ratio increases to some extent due to the usage of the semi-active H∞ control strategy. It is also found that an increase in the maximum permanent interstory drift ratio also occurs in both the semi-active MPC and Clipped-LQG strategies.

**Fig. 6:**
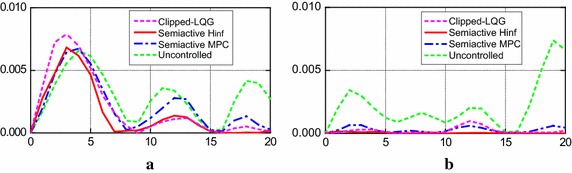
Distribution of permanent interstory drift ratio. **a** Full-scale Northridge; **b** full-scale Kobe

To further discuss this issue, Fig. 6 depicts the permanent drift ratio response for the full-scale intensity Kobe and Northridge earthquakes. Although the maximum permanent drift ratio from the first floor to the third floor of the structure is somewhat larger for the semi-active H∞ control strategy under full-scale Northridge earthquake, the permanent offset at each floor in the controlled structure is usually a small part of the uncontrolled structure. In other floors, the permanent drift ratio is reduced effectively. Note that the semi-active H∞ control strategy performs much better than the other two schemes. In the case of full-scale Kobe earthquake, the permanent offset of the controlled structure is generally greatly smaller than that of the uncontrolled structure throughout all floors, and the control effect of the semi-active H∞ control strategy on the permanent offset is a little better than that of the other two strategies. Additionally, the evaluation criteria *J*_4_ in Table 2 is above 1.0 for the full-scale Northridge earthquake because of the existence of the permanent offset.

## Conclusion

This paper concentrates on the development of a semi-active control system for nonlinear high-rise buildings. A novel semi-active H∞ control strategy is presented and evaluated. In this scheme, a modified Kalman–Bucy observer which is suitable for the proposed semi-active strategy is developed to obtain the state vector from the semi-active control force and acceleration feedback, taking into account of the effects of nonlinearity, disturbance and uncertainty of controlled system parameters by the observed nonlinear acceleration.

The proposed control strategy is applied to the nonlinear response control of a 20-story benchmark structure when subjected to earthquake excitation. The results indicate that the proposed semi-active H∞ control strategy provides much better control performance by comparison with the semi-active MPC and Clipped-LQG control systems and can effectively reduce the nonlinear response of the structure subject to earthquake-induced motions. It is worth noting that the permanent offset in the interstory drifts was usually significantly reduced in the controlled structure. The number of plastic hinges generated in the controlled structure during each earthquake is also significantly reduced when compared with that in the uncontrolled structure. Therefore, damage to high-rise buildings under strong earthquakes could be significantly minimized.
